# Viral etiology of severe acute respiratory infections in hospitalized children in Cameroon, 2011–2013

**DOI:** 10.1111/irv.12391

**Published:** 2016-05-09

**Authors:** Sebastien Kenmoe, Patrice Tchendjou, Marie‐Astrid Vernet, Suzie Moyo‐Tetang, Tatiana Mossus, Mohamadou Njankouo‐Ripa, Angeladine Kenne, Véronique Penlap Beng, Astrid Vabret, Richard Njouom

**Affiliations:** ^1^ Centre Pasteur of CameroonInternational Network of Pasteur InstitutesYaoundeCameroon; ^2^ Essos Hospital CenterYaoundeCameroon; ^3^ Département de BiochimieUniversité de Yaoundé 1YaoundeCameroun; ^4^ Virology ServicePôle de BiologieCHU de CaenCaenFrance

**Keywords:** Africa, Cameroon, etiology, severe acute respiratory infections, virus

## Abstract

**Background:**

Severe acute respiratory illness (SARI) is recognized as an important cause of morbidity, mortality, and hospitalization among children in developing countries. Little is known, however, in tropical countries like Cameroon about the cause and seasonality of respiratory infections, especially in hospitalized settings.

Objectives: Our study investigates the viral etiology and seasonality of SARI in hospitalized children in Yaounde, Cameroon.

**Methods:**

Prospective clinic surveillance was conducted to identify hospitalized children aged ≤15 years presenting with respiratory symptoms ≤5‐day duration. Demographic and clinical data, and respiratory specimens were collected. Nasopharyngeal samples were tested for 17 respiratory viruses using a multiplex polymerase chain reaction. The viral distribution and demographic data were statistically analyzed.

**Results:**

From September 2011 through September 2013, 347 children aged ≤15 years were enrolled. At least one virus was identified in each of 65·4% children, of which 29·5% were coinfections; 27·3% were positive for human adenovirus (hAdV), 13·2% for human respiratory syncytial virus (hRSV), 11·5% for rhinovirus/enterovirus (RV/EV), 10·6% for human bocavirus (hBoV), 9·8% for influenza virus (Inf), 6·6% for human parainfluenza virus (hPIV), 5·7% for human coronavirus (hCoV), and 2·3% for human metapneumovirus (hMPV). While hRSV showed seasonal patterns, hAdV and RV/EV were detected throughout the year and no evident temporal patterns were observed for the remaining viruses.

**Conclusion:**

Respiratory viruses were associated with a high burden of hospitalizations among children in Cameroon. Nevertheless, additional studies evaluating asymptomatic Cameroonian children will be important in understanding the relationship between viral carriage and disease.

## Introduction

Severe acute respiratory illness (SARI) is the leading cause of hospitalization, morbidity, and mortality in children younger than 5 years throughout the world. The burden of these infections is particularly important in developing countries with a meta‐analysis estimating that 265 000 (95% CI 160 000–450 000) in‐hospital deaths took place in 2010 with 99% of those deaths occurring in developing countries.^1^ Although there is little information on the causes of these respiratory illnesses in developing countries, available data indicate that viral agents play an important role globally.^2^ A meta‐analysis estimates that hRSV causes 33·8 million infections each year among children younger than 5 years. About 3·4 million of these cases require hospital admission, and an estimated 66 000–199 000 of children die with 99% of deaths occurring in developing countries.^3^ Many other viruses such as influenza viruses, rhinoviruses (RV), human parainfluenza viruses (hPIV), human adenoviruses (hAdV), human coronaviruses (hCoV‐OC43, hCoV‐229E, hCoV‐NL63, and hCoV‐HKU1), and enteroviruses (EV) have also been implicated worldwide.^2^, ^4^, ^5^ Discovered recently, human metapneumoviruses (hMPVs) and human bocaviruses (hBoVs) are viruses resulting in similar symptoms. Other emergent viruses such as SARS‐CoV and MERS‐CoV are responsible for more severe symptoms like respiratory distress syndrome.^6^, ^7^, ^8^ Real‐time reverse‐transcription polymerase chain reaction (rRT‐PCR) assays have been shown to be a sensitive and specific tool for the detection of these viruses. Furthermore, the introduction of multiplex rRT‐PCR enables the set‐up of assays detecting two or more targets in single clinical specimen.^9^, ^10^

In 2007, the Centre Pasteur du Cameroun (CPC) began implementing sentinel surveillance for influenza in Cameroon. This surveillance system has enabled a better understanding of the epidemiology of influenza in the country.^11^, ^12^ Even though our recent publication has demonstrated that those respiratory viruses play an important role in patients consulting our sentinel sites,^13^ there remains a lack of information regarding patients hospitalized with SARI.

This study was conducted to gain new insights into the timeliness of virus circulation and viral etiology among children aged ≤15 years, hospitalized for SARI.

## Materials and methods

### Study design

This prospective observational study was conducted in the pediatric service of ‘Centre Hospitalier d'Essos’ in Yaounde, Cameroon. This central region has an equatorial climate with a defined moderate rainfall during March–June and intense precipitation during September–November.

### Subjects

Children aged less than or equal to 15 years hospitalized for SARI were daily recruited in our study. A total of 347 patients were enrolled by experienced nurses or physicians during a 2‐year period between September 2011 and September 2013. SARI was defined according to a previously suggested WHO case definition^14^ with a history of symptoms of <5 days. These criteria of hospitalized patient inclusion were as follows: fever >38°C and cough and/or sore throat.

### Ethical considerations

Children were enrolled only after an informed written consent was obtained from their parents or legal guardians. This study was part of a global study aimed at assessing risk factors associated with severe influenza in Cameroon (IMMI Project), which was reviewed and approved by the National Research Ethics Committee and the Ministry of Health of Cameroon.

### Study and laboratory procedures

Demographic and clinical information was obtained from participants using a standardized questionnaire.

Nasopharyngeal swab specimens were collected from hospitalized children and transported to the CPC as previously described.^13^ Viral RNA and DNA were extracted using QIAamp Viral RNA Mini kit and QIAamp DNA Mini kit (Qiagen, Hilden, Germany), respectively, following the manufacturers' instructions. A final elution volume of 60 μl of RNA and 200 μl of DNA were stored at –80°C till testing by multiplex PCR/RT‐PCR. Testing for influenza A and B viruses were conducted, and influenza A virus‐positive specimens were subtyped according to the method developed by US Centers for Disease Control and Prevention (CDC).^15^ Then, the detection of 14 non‐influenza respiratory viruses, including hMPV, hRSV, hPIV (1–4), EV, RV, hCoV‐OC43, hCoV‐229E, hCoV‐NL63, hCoV‐HKU1, hAdV, and hBoV were performed by 4 duplex rRT‐PCR, using a commercially available Respiratory Multi Well System r‐gene^™^ (bioMérieux, Lyon, France) following the manufacturer's instructions. PCR amplifications were performed on ABI 7500 Fast (Applied Biosystems, Foster City, CA, USA). A patient was considered to have a single viral infection if only one pathogen was detected in the specimens. In case of more than one viral pathogen detected, patients were considered to have viral coinfections.

### Statistical analysis

The data obtained and the laboratory results were anonymized and entered into a database prepared with Microsoft^®^ Excel (Microsoft, Washington, DC, USA). The database was then checked and cleaned for abnormal wrongful entries. We analyzed the demographics of the study subjects and the positive cases, as well as the clinical characteristics of all respiratory viruses. Proportions were compared using a chi‐squared or the Fisher exact test. We analyzed the clinical and coinfection characteristics of positive according to the negative patients for respiratory viruses using a stepwise logistic regression model. Uncertainty was expressed as 95% confidence intervals (CIs). Statistical analysis was performed with the R program version 2·15·1, and *P*‐values of 0·05 or less were considered statistically significant.

## Results

### Demographic characteristics

During the study period, 374 hospitalized children with SARI aged 0–15 years were enrolled. As 27 patients were excluded due to insufficient material for analysis of all viruses, 347 (92,8%) subjects were analyzed. The median age of the patients was 1·5 years (IQR: 0·75–3·0 years). Most patients (88·4%) were below 5 years of age, with 131 (37·8%) children ≤1 year, 100 (28·8%) children aged [1–2] years, 76 (21·9%) children aged [2–5] years, and 40 (11·5%) children aged [5–15] years. Half of patients (180 [51·9%]) were male. Among the 347 hospitalized participants with SARI, the most common respiratory symptoms were cough (85·9%, 298/347), rhinorrhea (78·4%, 272/347), fatigue (57·9%, 201/347), and wheezing (41·8%, 145/347). Demographic data and clinical characteristics of the 347 cases studied are summarized in Table 1.

**Table 1:** Demographic and clinical characteristics of all participants (*N =* 347)

Characteristics	SARI recruited (*n* = 347)	Negative samples (*n* = 120)	Respiratory viruses detected		
			Total (*n* = 227)	1 (*n* = 160)	>1 (*n* = 67)
Sex					
Male	180 (51·9)	61 (50·8)	119 (52·4)	85 (53·1)	34 (50·8)
Female	167 (48·1)	59 (49·2)	108 (47·6)	75 (46·9)	33 (49·2)
Age					
≥ 1 Year	131 (37·8)	43 (35·8)	88 (38·8)	59 (36·9)	29 (43·3)
[1–2] years	100 (28·8)	32 (26·7)	68 (30·0)	50 (31·2)	18 (26·9)
[2–5] years	76 (21·9)	26 (21·7)	50 (22·0)	35 (21·9)	15 (22·4)
[5–15] years	40 (11·5)	19 (15·8)	21 (9·2)	16 (10·0)	5 (7·4)
Symptoms					
Cough	298 (85·9)	99 (82·5)	199 (87·7)	137 (85·6)	62 (92·5)
Rhinorrhea	272 (78·4)	89 (74·2)	183 (80·6)	129 (80·6)	54 (80·6)
Fatigue	201 (57·9)	60 (50)	141 (62·1)	104 (65·0)	37 (55·2)
Wheezing	145 (41·8)	49 (40·8)	96 (42·3)	67 (41·9)	29 (43·3)
Vomiting	131 (37·8)	42 (35)	89 (39·2)	63 (39·4)	26 (38·8)
Diarrhea	100 (28·8)	37 (30·8)	63 (27·8)	46 (28·8)	17 (25·4)
Sore throat	75 (21·6)	39 (32·5)	36 (15·9)	26 (16·3)	10 (14·9)
Headache	46 (13·3)	23 (19·2)	23 (10·1)	21 (13·1)	2 (3·0)
Conjunctivitis	36 (10·4)	12 (10)	24 (10·6)	185 (11·3)	6 (9·0)
Arthralgia	29 (8·4)	12 (10)	17 (7·5)	16 (10·0)	1 (1·5)
Cutaneous rash	26 (7·5)	11 (9·2)	15 (6·6)	14 (8·8)	1 (1·5)
Myalgia	17 (4·9)	10 (8·3)	7 (3·1)	6 (3·8)	1 (1·5)
Earache	13 (3·7)	5 (4·2)	8 (3·5)	6 (3·8)	2 (3·0)

Data are number and percentage in brackets.

### Clinical characteristics and viral agents identified

**Figure 1:**
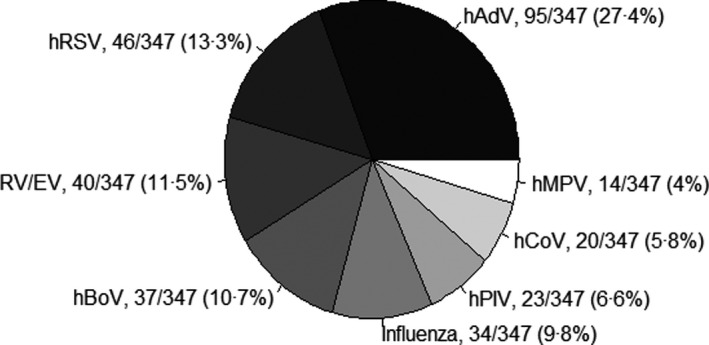
Detection rate of each virus or group of viruses in 347 respiratory samples. Positive cases are the sum of single and mixed infections for each virus or group of viruses.

Respiratory viruses were observed in 227 [65·4%; 95%CI: 60·1–70·4] of the 347 samples analyzed for the 17 respiratory viruses tested. The most frequently identified pathogen was hAdV (27·4%, 95/347) followed by hRSV (13·3%, 46/347), EV/RV (11·5%, 40/347), hBoV (10·7%, 37/347), influenza (9·8%, 34/347; consisted of influenza A/H3N2 [6·3%, 22/347], influenza B [2·3%, 8/347], influenza A/H1N1/2009 [1·2%, 4/347]), hPIV (6·6%, 23/347), hCoV (5·8%, 20/347), and hMPV (4%, 14/347) (Figure 1).

A majority of SARI was observed among children aged ≤5 years (90·7%, 206/227), and the proportions infected were significantly negatively associated with age (odds ratio [OR], 0·91; 95% CI, 0·84–0·99, *P* = 0·03). There were no significant differences in terms of gender between patients infected with different viruses. Influenza virus were more frequently detected in the individuals over 1 year (*P* = 0·04). For the remaining viruses, there was no difference between rate of infection and age (Table 2).

**Table 2:** Detection rate and age‐specific distribution of each virus or group of viruses detected and rate of coinfection

	Total	[0–1] year	[1–2] years	[2–5] years	[5–15] years	*P*‐value
N	347	131	100	76	40	
HAdV	95 (27·4)	40 (30·5)	27 (27)	20 (26·3)	8 (20)	0·61
HRSV	46 (13·3)	20 (15·3)	11 (11)	12 (15·8)	3 (7·5)	0·47
RV/EV	40 (11·5)	16 (12·2)	15 (15)	7 (9·2)	2 (5)	0·37
HBoV	37 (10·7)	16 (12·2)	9 (9)	8 (10·5)	4 (10)	0·88
Influenza	34 (9·8)	6 (4·6)	15 (15)	9 (11·8)	4 (10)	0·04
HPIV	23 (6·6)	9 (6·9)	4 (4)	8 (10·5)	2 (5)	0·39
HCoV	20 (5·8)	8 (6·1)	5 (5)	4 (5·3)	3 (7·5)	0·94
HMPV	14 (4·0)	5 (3·8)	6 (6)	0 (0)	3 (7·5)	0·14
Coinfection	67 (29·5)	29 (33)	18 (26·5)	15 (30)	5 (31·3)	0·76
Any viral infection	227 (65·4)	88 (67·2)	68 (68)	50 (65·8)	21 (52·5)	0·33

hAdV, human adenovirus; hRSV, human respiratory syncytial virus; RV/EV, rhinovirus/enterovirus; hBoV, human bocavirus; hPIV, human parainfluenza virus; hCoV, human coronavirus; hMPV, human metapneumovirus.

Data are number and percentage in brackets.

**Figure 2:**
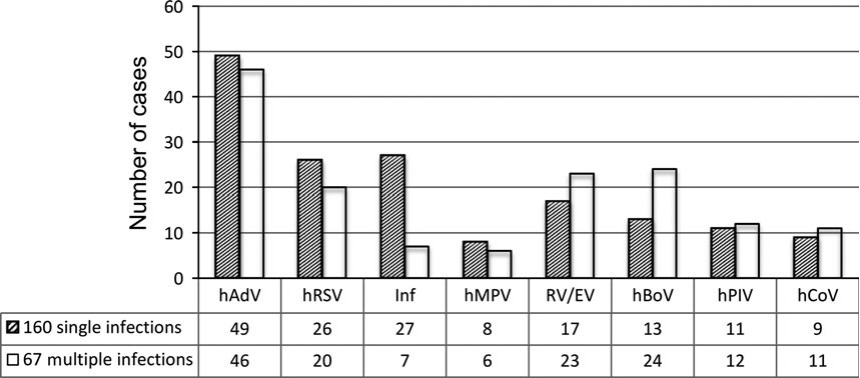
Number of each respiratory virus/group of viruses detected as single or mixed infections.

Among positive samples, single viral infection accounted for 70·5% (160/227) of cases, whereas infections with multiple viruses were observed in 29·5% (67/227), including 53 double infections, 13 triple infections, and one quadruple infection. Specific viruses detected in the study population of 347 hospitalized children are listed in Table 3, in descending detection rate. The mean age and age group distribution of patients with multiple viral infections were not significantly different from those of group with single infection (Table 2). HAdV, hRSV, Influenza and hMPV were more frequent in single infections whereas RV/EV, hBoV, hPIV, and hCoV were more frequent in mixed infections (Figure 2).

**Table 3:** Contribution of respiratory viruses to severe acute respiratory illness among hospitalized patients in Cameroun as monoinfection or coinfection

	AdV	RSV	RV/EV	hBoV	PIV	A/H3N2	hCoV	hMPV	Inf B	A/H1N1p	Total (*N =* 225)
AdV	49	9	8	9	4	1	2	2	0	0	95 (27·3)
RSV		26	2	1	0	3	1	0	0	0	46 (13·2)
RV/EV			17	4	0	0	1	1	0	0	40 (11·5)
hBoV				13	0	1	0	1	0	0	37 (10·6)
PIV					11	0	1	0	0	0	23 (6·6)
A/H3N2						16	0	0	0	0	22 (6·3)
hCoV							9	0	1	0	20 (5·7)
hMPV								8	0	0	14 (4·0)
Inf B									7	0	8 (2·3)
A/H1N1p										4	4 (1·1)
1 Virus	49	26	17	13	11	16	9	8	7	4	160 (70·5)
2 viruses	35	16	16	17	6	5	6	4	1	0	53 (23·3)
3 Viruses	10	4	6	7	5	1	4	2	0	0	13 (5·7)
4 Viruses	1	0	1	0	1	0	1	0	0	0	1 (0·4)

hAdV, human adenovirus; hRSV, human respiratory syncytial virus; RV/EV, rhinovirus/enterovirus; hBoV, human bocavirus; hPIV, human parainfluenza virus; hCoV, human coronavirus; hMPV, human metapneumovirus.

Data are number and percentage in brackets.

In a binary logistic regression, positive samples was negatively associated only with headaches (*P* = 0·003, odds ratio [OR], 0·08; 95% CI, 0·01–0·3). No other clinical symptoms were associated with the positive patients, nor with any other specific viruses detected or coinfections.Seasonality.

The study also investigated the monthly distribution of viruses. The seasonal variations of the viral respiratory infections are shown in figure 2. In the first period from September 2011 to November 2012, the average number of samples collected monthly was 18·7, whereas in the second period from December 2012 to September 2013 the number of enrolled cases was much lower, the average number of samples collected monthly being 6·6. However, hRSV showed a pronounced seasonality, with peaks during the rainy season from September to December. The circulation of influenza viruses was similar to hRSV although a few cases were detected outside the rainy season peak. RV/EV was detected almost year‐round with peak activity in the rainy season between September and December corresponding to a higher number of samples collected during that period. On the other hand, hAdV occurred throughout the study period with no evident temporal patterns. hBoV occurred intermittently all over the study period, while the prevalence of remaining viral etiologies was low, ranging from 0 to 5 positive samples per month for each virus, numbers which appeared insufficient to allow for the description of seasonal patterns. Coinfections were observed throughout the study period.

**Figure 3:**
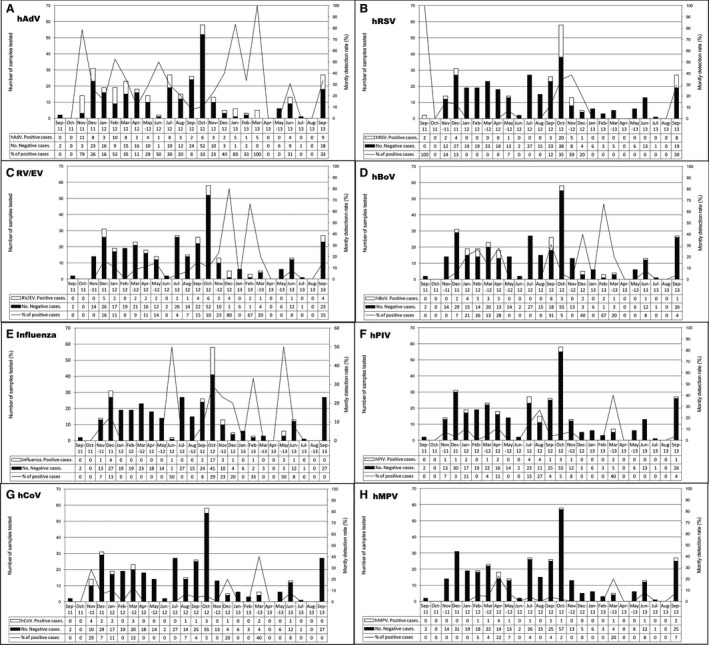
Seasonal distribution of respiratory viruses or group of viruses detected in children with SARI during September 2011 till September 2013. Primary *y*‐axis and bars describe the number of cases, while secondary *y*‐axis and lines describe the monthly detection rate.

## Discussion

The aim of this study has been to determine the viral panorama of SARI in 374 hospitalized children during a 2‐year period in Cameroon. The data presented here are unique, to the best of our knowledge. It represents viral etiologies (17 respiratory viral targets) in children aged ≤15 years hospitalized for SARI in Cameroon. More importantly, this study describes the incidence of AdV and the recently discovered hBoV in Cameroon for the first time.^8^ Our results demonstrate the potential importance of respiratory viruses in the etiology of hospitalized children with SARI in Cameroon.

Of 347 samples tested, at least one respiratory virus was detected in 227 (64·5%). This detection rate is consistent with results of other etiologic studies of hospitalized children conducted in similar settings.^16^, ^17^, ^18^, ^19^, ^20^ Nonetheless, this proportion appears to vary largely between countries. Kwofie *et al*. recorded a lower percentage of 25·7% in Ghana^21^ and Khor *et al*. recorded 26·4% in Malaysia.^22^ Higher percentages 94·2% in China,^23^ 91% in France,^24^ and 90% in Alaska^25^ were reported by Zhang *et al*., Singleton *et al*., and Huguenin *et al*., respectively. Comparison among published studies is always difficult. Possible reasons for these varying outcomes among studies include true differences in epidemiology, differences in the inclusion criteria for study population, diagnostic methods used, panel of viral agents tested, climate, study duration, and patients' ages. Consider that all the above studies included hospitalized children; lower prevalence recorded by Khor *et al*. might have been due to their 27‐year study duration and laboratory methods (direct IF and viral isolation) which are known to be less sensitive than molecular techniques.^25^

The most prevalent virus detected in our study was AdV, representing 27·3% of the total number of viruses observed. This value is higher than that (0·8–13·3%) reported in most other similar studies.^16^, ^17^, ^22^, ^26^, ^27^, ^28^ It is nonetheless similar to what was noted in Alaska^24^ and in Mozambique^29^ by Singleton *et al*. (30%) and O'Callaghan‐Gordo *et al*. (21%). The serotypes B(3,7,21) and E(4) of AdV have been implicated in more severe diseases including bronchitis or pneumonia^30^ in the immunocompromised host and children particularly. This finding shows that AdV could play a more significant role than originally thought. Accordingly, additional efforts describing our associated serotypes will help to further understand how much this virus contributes to disease severity.

Unlike our results, most similar studies showed that hRSV was the most prevalent viral pathogen.^22^, ^31^, ^32^, ^33^ In our study, hRSV was the second most frequent pathogen after hAdV with merely 13·2% of 347 hospitalized children testing positive. However, this proportion was similar to that reported in other studies (5·7–15%).^16^, ^28^, ^29^, ^34^ Unlike previous similar studies, RV/EV accounted for only 11·5% of positivity in our study.^17^, ^18^, ^24^, ^26^, ^29^ HBoV was the fourth most common virus with a prevalence of 10·6% in all cases. This result is in line with the results of others authors, who have reported a worldwide detection rate of hBoV ranges between 2 and 19% in nasopharyngeal samples.^35^ The remaining viral etiologies were identified in 9·8%, 6·6%, 5·7%, and 2·3% for influenza, HPIV, HCoV, and HMPV, respectively. These results correspond with those reported by other authors.^23^, ^27^, ^32^, ^33^, ^36^, ^37^

As previously shown, we also found that virus‐associated hospitalization figures were significantly higher among children (*P* = 0·03).^27^, ^28^, ^38^ According to reports, there is always a higher incidence of viral respiratory infection in children than in adults. This may be partially explained by the lower detection rate of respiratory viruses in the elderly due to reduced viral shedding in older age groups.^39^ HRSV, the second most commonly virus detected in this study, has been reported to be the leading cause of SARI in young children.^19^, ^22^, ^36^ Nevertheless, we did not find a significant age‐specific prevalence in HRSV detection during this study. This was probably due to the fact that our statistical analysis was hampered by the limited size of study participants older than 5 years. Headaches were negatively associated with positives samples (*P* = 0·003). However, as a majority of our study population was made up of children unable to report headaches, only 46 patients (13·3%) were taken into account in the analysis.Multiple viral infections were observed in 29·5% of the patients with positive viral detection. This is a very similar percentage to the one found by others researchers.^22^, ^26^, ^37^ Bocavirus has been frequently implicated in coinfections.^17^, ^19^, ^25^ Our results reflect those of other studies in which hBoV was associated (35·8% versus 8·1%, *P* < 0·001) with multiple infections. Surprisingly, AdV and picornaviruses were also most frequently involved in coinfections, which probably resulted from the fact that they were detected throughout the year and were among the most prevalent.

In spite of our intensive surveillance strategy with daily recruitment, the number of inclusions was not stable over the study period. Notwithstanding this lack of uniformity in number of samples collected during the first (September 2011 to November 2012) and second (December 2012 to September 2013) periods, we were able to detect a seasonal pattern thanks to the sufficient number of enrolled patients during two consecutive years. We found that RSV cases were detected mainly during the major rainfalls from September to November, which is consistent with the data in the literature.^22^, ^23^, ^31^ The seasonality of influenza virus is very uncertain in tropical areas. The current study did not confirm that influenza circulated between September and November, as shown in our previous studies.^13^ This fact might have contributed to the reduction in the inclusion rate during the second period due to a true change in the epidemiology of SARI. Indeed, that would have contributed to reduce the numbers eligible patients in the second period of the study. RV/EV and AdV circulated year‐round, a finding reported in other studies.^17^, ^23^, ^28^ For the remaining viruses, no evident temporal patterns were observed. This study is ongoing, however, and any changes in the seasonal circulation will be detected.

Several points must be taken into consideration when looking at our findings. Firstly, our study was conducted at only one site, which might have underestimated the overall detection rate for selected viruses. Likewise, the enrolled cases may not be generalizable for the entire population of children in Cameroon. Subsequently, as discussed by Broor *et al.,* 38 there is no denial that some respiratory viruses can be detected in asymptomatic children^24^, ^40^, ^41^ or shed for long periods of time after infection.^24^, ^42^ Hence, the role played by the viruses detected in these SARI cases is less clear considering that we did not evaluate respiratory specimens from asymptomatic children without respiratory symptoms. Otherwise, despite tests for a large panel of respiratory viruses we did not evaluate bacterial etiologies, due to the difficulty in obtaining adequate samples for culture. It has been shown that bacterial pathogens may have been responsible for SARI symptoms^16^; further studies will thus be required to specify their role in SARI in Cameroon. Lastly, although nurses in charge of recruitment were well trained, they did not systematically record severe symptoms such as convulsion, stridor, chest pain, inability to drink or breastfeed, and outcomes of study participants. This limited our ability to assert the clinical case severity and outcome of our SARI cases. This situation also added a limitation to this study regarding the representativeness of children included.

Notwithstanding its limits, this is the first study in Cameroon to characterize 17 common respiratory viruses in pediatrics with SARI during a 2‐year consecutive period. Using a multiplex rRT‐PCR, we were able to identify a wide variety of viruses and their seasonal patterns. In our resource‐limited setting, this process is particularly useful for selecting only the major contributors to SARI and thereby enhancing our existing surveillance systems. Additional studies evaluating asymptomatic Cameroonian children will be important to understanding the relationship between viral carriage and disease. With this new method, we will also be able to evaluate the role of viral loads in respiratory infections in future research.
